# Appendicitis

**Published:** 1893-09

**Authors:** James Lilienthal

**Affiliations:** San Francisco, Cal.


### APPENDICITIS.

James Lilienthal, M. D., San Francisco, Cal.

During the last two years, I doubt if any special disease has been the subject of so many articles in our journals, or the cause of so much discussion in the various societies of our country and Europe as the subject of my present paper. Doubts, on the one hand, of many of our older practitioners as to the frequency of its occurrence, well illustrated by a remark of Dr. Wyman, in an article in the New York *Medical Journal*, of February 25th, who states that he was asked: "Doctor, how is it that you surgeons find so many cases of what you call appendicitis to operate upon?" but, more than all, the question of treatment, medicinal or surgical, is the bone of contention.

Our surgeons, no matter of what school, believe that the knife, and the knife alone, is the proper method of treatment, and, with that disregard of the dangers of incising the peritoneum with which their frequent abdominal operations have imbued them, they see little or no danger in the operation; if, unfortunately, the case terminates fatally, they lay the blame on the poor physician who wasted valuable time, and only called them in when sepsis had already taken a firm hold of its victim. Without for a moment wishing to decry the necessity of operative measures, which at times are essential, and the only hope for the patient, the question necessary to be considered is, when is the time that internal medication must give place to surgery?

We have all seen cases recover after the surgeon had stated that the only chance was in an operation, and, *vice versa*, patients pass away because physical examination had failed to demonstrate the presence of pus, only the *post-mortem* examination teaching us that a laparotomy would have given the patient a chance for life.

We must not, however, forget that the internal treatment of the two schools makes a material difference in the picture presented to our view. While the old school hold firmly to the use of opiates, and thereby mask the symptoms of the disease, becoming only aware of its progress when a general peritonitis, due to a rupture of the gut, is upon them, I claim that with our remedies we are less liable to have this danger spring upon us, and can turn the case over to the surgeon while the patient is yet in a good condition to undergo the shock of a surgical operation. Just when this step is necessary is the point that I should like to have discussed by my colleagues, so that one branch of our art may supplement the other at the proper time, and for the good of the lives intrusted to our care.

The symptoms of the disease are too well known to warrant their recital, especially as they will necessarily appear to some extent in the symptomatology of the various drugs; hence, we may pass at once to the treatment, giving, however, only the symptoms referring to the region affected, and omitting those well-known characteristics of the various drugs which are symptomatic alone of the drug, and are present whenever said drug is indicated.

In presenting the remedies that may be called for, it is needless for me to remark that *any* drug in our pharmacopœia may be the simillimum, but those most frequently required will be in the first stage:

*Aconite.*—Abdomen burning hot, tense, tympanitic, sensitive to the least touch; cutting pains; meteorism; vomiting; inability to urinate.

*Arnica.*—May be called for when trouble arises from traumatism; right side of abdomen hard swollen, with pain as if cutting into a wound when touched.

*Arum-maculatum.*—Painful spot in abdomen between navel and groin, especially when standing or lying on the side or back, most when expanding the chest or stretching the abdominal muscles; painful to external pressure.

*Belladonna.*—Great pain in ileo-cœcal region; cannot bear the slightest touch, not even of bed-cover; worse by the least jar or turning of the body, so lies immediately on his back.

*Calendula.*—Constant pains in abdomen and sensitiveness in right iliac region; dull, coarse stitches, worse during movement, disappearing during rest.

*Camphor.*—Drawing, bruised pain, more internally than externally, especially on inspiration, in whole right side of abdomen, extending into region of liver and into chest.

*Carduus-marianus.*—Awakens with pain in abdomen near right hip bone; pressing, stitching pains; distention of abdomen on right side; sensation of motion in intestines on expiration.

*Cocculus.*—Constant pain in right iliac region, near cœcum; worse from least touch; pain often remits, returning, however, very much aggravated; during paroxysm, drawing pains through whole abdomen, causing her to work her limbs constantly; can find relief in no position.

*Doryphora.*—Abdomen sore and heavy; violent pains in right side, passing downward to rectum.

*Ferrum-phosphoricum.*—Dr. Nicholson, of Oakland, refers to a cure with this remedy, in alternation with Kali-muriaticum. The symptoms were agonizing pains in right iliac region, swelling in this region exquisitely tender to touch, with high fever, vomiting, etc.

*Inula* (*Elecampane*).—Sticking, as from a knife, between umbilicus and right groin; violent motion beneath right hypochondrium.

*Rhus-toxicodendron.*—Hard, painful swelling of nearly the entire right side of abdomen; pain worse on sitting or when stretching right leg; impossibility of lying on left side; better when lying on back with knees drawn up, or when gently pressing the swelling from below upwards.

Should no improvement be manifested, and the symptoms evidence beginning suppuration, we may use:

*Apis.*—Beginning of exudative stage; burning, stinging in abdomen, with great tenderness to touch and on turning in bed; pains extending into thighs or upwards to the ribs.

*Baptisia.*—Right iliac region sensitive; abdominal muscles sore to pressure, with acute intermittent pain; stitches in right groin, coming and going.

*Bryonia.*—Pain on a limited spot in abdomen, dull, with a throbbing sensation; abdomen very sore to touch; accompanied by constipation, and worse by standing and motion.

*Hepar.*—Deep, circumscribed swelling in ileo-cœcal region; lies on back, with right knee drawn up.

*Lachesis.*—Lies on back, with knees drawn up; tenderness, pain, and swelling in region of cœcum; pain, extending from right lumbar region through sacrum, through inguinal region and fore part of thigh, more especially when swelling is examined; urine scanty, high colored, strangury; bowels costive.

*Mercurius-solubilis.*—Painful, hard, hot, and red swelling in ileo-cœcal region, painful to touch, preventing extension of thigh; must lie upon back, with thigh flexed; fever, flushed face; red, dry tongue.

*Mercurius-corrosivus.*—Peculiar bruised sensation in abdomen, especially in cœcal region, and following course of transverse colon; painful to moderate pressure, as though bruised.

*Plumbum.*—Distention and hardness of cœcum and ascending colon, whole region swollen, painful to touch, on motion; tongue dry, brown in centre; feeling of lameness in lower extremities.

It is this stage that requires our greatest watchfulness. The remitting fever, the dry tongue, either red or furry; the apathetic condition, possibly chills—in one word, the typhoid condition—warns us of impending suppuration, and the life of our patient depends upon our judgment as to the length of time that we may wait before urging upon the family the danger of delay and the absolute necessity of surgical interference; for, although recovery may take place after perforation has occurred, we well know that the danger has become multiplied by such an accident. We find cases, however, where, in spite of our best advice, supplemented, perhaps, by that of counsel, consent to surgical measures cannot be gained. In these cases we must rely on the following remedies, which may still prove effectual, and we will have the satisfaction of knowing that we have done all in our power to obtain a satisfactory result:

*Arsenicum.*—Abdomen enormously distended; burning lancinations in abdomen, with great restlessness, which, however, aggravate the pains; sudden sinking of strength, cold sweat.

*Crotalus.*—Extreme pain in region of appendix, with frequent paroxysmal aggravations, great tenderness on pressure in a spot of the size of a small orange, with some feeling of hardness; cannot bear right leg straightened, but lies with it bent and supported by a pillow; skin hot and dry.

*Opium.*—General peritonitis; face flushed, skin hot and damp, peristaltic motion of intestines, reversed or paralyzed; belching and vomiting.—*From The Pacific Coast Journal of Homœopathy, June,* 1893.

